# Detection of rabies virus in *Callithrix penicillata* (Geoffroy, 1812) in Montes Claros, Minas Gerais State, Brazil

**DOI:** 10.1590/0037-8682-0029-2024

**Published:** 2024-07-29

**Authors:** Milton Formiga Souza, Thallyta Maria Vieira, Agna Soares da Silva Menezes, Maria Clara Lélis Ramos Cardoso, Dulce Pimenta Gonçalves, Vanessa Ferreira da Silva, Gilberto Ramalho Pereira, Ronnie Antunes de Assis

**Affiliations:** 1 Secretaria Estadual de Saúde de Montes Claros, Montes Claros, MG, Brasil.; 2 UNIMONTES, Departamento de Biologia Geral, Montes Claros, MG, Brasil.; 3 Prefeitura Municipal de Montes Claros, Centro de Controle de Zoonoses, Montes Claros, MG, Brasil.; 4 UNIMONTES, Departamento de Ciências Agrárias, Janaúba, MG, Brasil.

**Keywords:** Rabies virus, Chiropteran (*Artibeus sp*), Marmoset (*Callithrix penicillata*)

## Abstract

This report describes the occurrence of the rabies virus in two species of wild animals in the urban area of Montes Claros (MOC), Minas Gerais State, Brazil, in May 2023. The virus has been detected in frugivorous chiropterans (*Artibeus sp*) and marmosets (*Callithrix penicillata*). This is the first notified case of the rabies virus in the species *C. penicillata* in the urban area of MOC. Our findings show that the rabies virus is circulating in the urban area of MOC; therefore, permanent preventive measures must be adopted to avoid infection of other animals and humans.

## INTRODUCTION

Rabies is a zoonotic disease caused by infection with a virus belonging to the *Lyssavirus* genus. It affects the central nervous system of mammals and has a mortality rate of ~100%^1^. Approximately 60,000 human deaths are recorded per year worldwide^2^. 

Seven genotypes of the rabies virus are known: the classic rabies virus (RABV type 1), Lagos Bat virus (LBV - type 2), Mokola virus (MKV - type 3), Duvenhage virus (DV - type 4), European Bat virus 1 (EBLV1 - type 5), European Bat virus 2 (EBLV-2 - type 6), and Australian Bat virus (ABLV - type 7)^3^. RABV type 1 is the most common RABV genotype in Brazil. The variants were identified among these genotypes. The variants AgV1 and AgV2 are predominantly isolated from dogs, AgV3 is associated with the hematophagous bat *Desmodus rotundus;* AgV4 and AgV6 are isolated from insectivorous bats, such as *Tadarida brasiliensis* ^4^.

The most effective strategies for preventing and controlling rabies involve a combination of measures, including annual mass vaccination campaigns for dogs and cats, health education of the population, and post-exposure treatment with vaccines and/or human anti-rabies serum for people who have suffered aggression from suspected animals. Currently, the most significant records of rabies cases in wildlife animals in Brazil are caused by bats, both hematophagous and non-hematophagous, as well as wild canids and marmosets^5^.

The rabies virus has four transmission cycles: wild, air, urban, and rural. Since 2004, the air and the wild cycles have been signaling a growing increase in the proximity of wild species to humans as a result of various factors, such as deforestation, the option of keeping them as pets in the houses, among others^6^.

In Brazil, there are important wild reservoirs of the rabies virus, including the wild canid *C. thous*, marmoset *Callithrix jaccus*, hematophagous bats (*Desmodus rotundus*), and a diversity of non-hematophagous bats adapted to urban environments, representing a potential risk of rabies transmission^7^.

In the Brazilian Cerrado biome (tropical savanna ecoregion), it is possible to observe several species of non-human primates (NHP), particularly the marmoset (*Callithrix penicillata*), a species classified in 1812 (Geoffroy, 1812). *C. penicillata* is native to the Brazilian Cerrado (tropical savanna ecoregion), has black tufts on the ears, and is present in the following Brazilian regions: south of the Amazon, Bahia, Goiás, Minas Gerais, and North of São Paulo and Rio de Janeiro states^8^. It is a species that, in general, lives in groups of two to 15 animals^9^. Their habitats are at the top of trees, and they feed on fruits, seeds, invertebrates, small vertebrates, eggs and tree gums^10^.

Therefore, we report the first case of rabies in an NHP from *C. penicillata* in the urban area of Montes Claros, Minas Gerais State, Brazil. A review of specialized literature was performed using the Cochrane Library, LILACS, SciELO, MEDLINE, PubMed, and PubMed Central (PMC) databases.

## CASE REPORT

**FIGURE 1:**
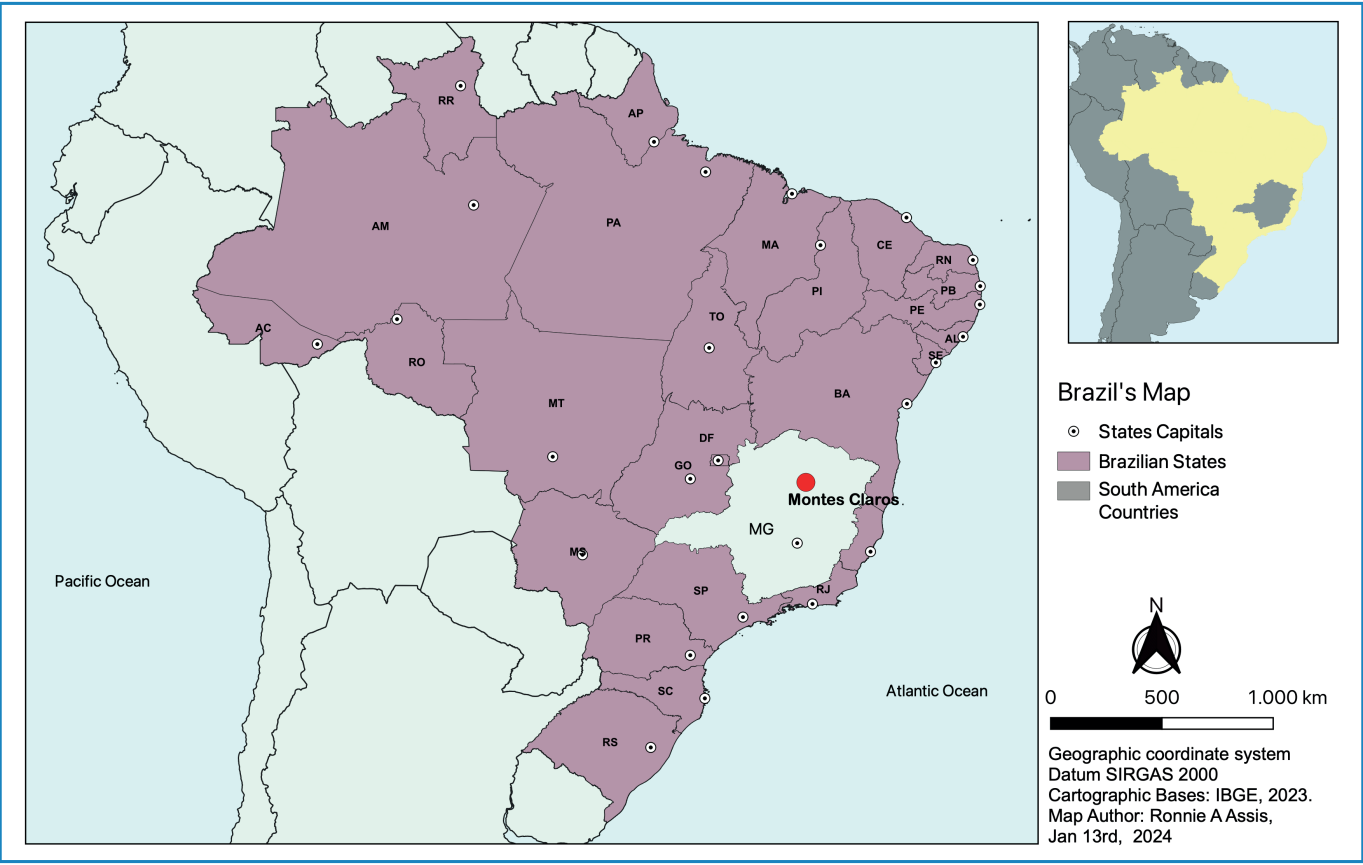
Map of Brazil showing the index case of rabies in a marmoset (*Callithrix penicillata*) in Montes Claros city, Minas Gerais State, Brazil.

This case was reported in May 2023 in an urban area of Montes Claros City (MOC), Minas Gerais, Brazil. MOC (latitude: -16, longitude: -43, altitude: 661 m) is the largest city in northern Minas State, with over 400,000 inhabitants, and is located in the Brazilian Cerrado biome (tropical savanna ecoregion) (Figure 1 **)**.

**FIGURE 2:**
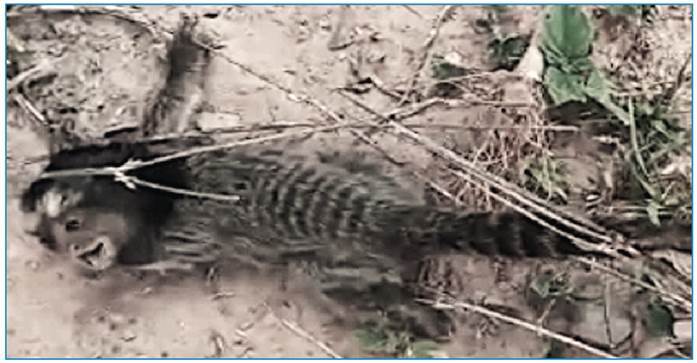
*Callithrix penicillata* that was found dead in Montes Claros city.

**FIGURE 3:**
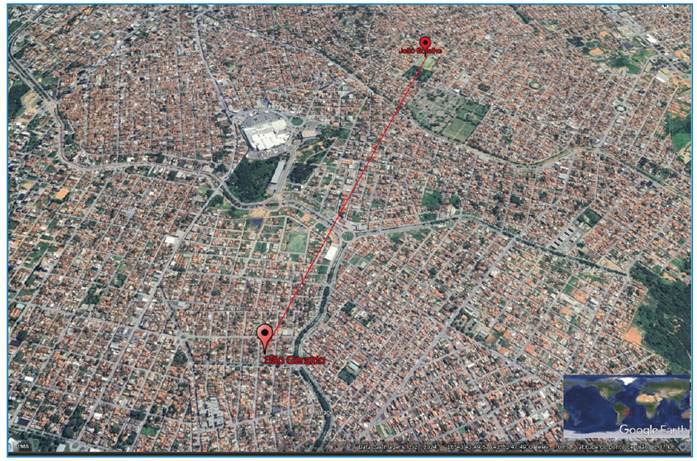
Map showing the distance between the rabies case in a marmoset (*Callithrix penicillata*) (São Geraldo II neighborhood) and the bat case (João Botelho neighborhood) in the urban area of Montes Claros city, Minas Gerais State, Brazil.

A female adult black-tufted marmoset (*C. penicillata*) weighing 200 g, with a body length of 24 cm and tail length of 30 cm, was found dead in an urban area of the city in the São Geraldo II neighborhood by a team from the Zoonosis Control Center of the MOC (CCZ-MOC). After performing a full necropsy, the central nervous system (CNS) was sent to the Zoonosis Laboratory of the City Hall of Belo Horizonte-MG-Brazil (Lzoon-PMBH), under refrigeration (2-8 °C), according to the conservation and shipping guidelines recommended by the Institution^11^. Furthermore, in the same week, *C. penicillata* was found dead (Figure 2), and a frugivorous bat (*Artibeus sp*) was also found dead in another neighborhood of the MOC (João Botelho) (Figure 3) and was sent to Lzoon-PMBH under the same conditions described for *C. penicillata*.

Using CNS sections from both animals, a direct fluorescent antibody technique (DFAT) and a mouse inoculation test (MIT) were performed at Lzoon-PMBH to diagnose rabies. Both assays yielded positive results. Fragments from the CNS of each animal were sent separately under refrigeration to the Pasteur Institute, São Paulo State, Brazil, to undergo molecular tests for rabies, including reverse transcription, Real-Time Polymerase Chain Reaction (RT-PCR), and sequencing. Two animals tested positive for the rabies virus.

After these results, CCZ-MOC promoted rabies prevention in animals and humans, including dog vaccination boosters and health education.

## DISCUSSION

*C. penicillata* is one of the most commonly identified wildlife species in the MOC region. It is present in both peri-urban and urban areas and is frequently observed in the early morning and late afternoon. There are few reports on the occurrence of rabies virus in NHP in Brazil. Cases were reported in the states of Ceará and Pernambuco up to 2012^7^ ^,^ ^12^ ^,^ ^13^, in Piauí since 2013^13^, in Bahia since 2017 (*D. rotundus* antigenic variant, AgV3)^13^, in Rio Grande do Norte since the late 1980s^12^ in *C. jacchus*, as well as, in Rio de Janeiro since 2019 (AgV3), in *Callithrix sp* ^14^; in Mato Grosso state, in *Cebus apella* ^15^ and Sergipe state, in *Callithrix spp* ^16^. Notably, in previous reports involving the *Callithrix* genus^14^ ^,^ ^16^, the authors did not report the species of *Callithrix* affected, but only the genus. Favoretto et al^7^ described rabies in *C. jacchus*. *C. jacchus* can be differentiated from *C. penicillata* based on the color of the tufts on the ears, which are white and black, respectively. Therefore, to the best of our knowledge, after a literature search of the Cochrane Library, LILACS, SciELO, MEDLINE, PubMed, and PubMed Central databases, we conclude that this is the first description of rabies virus in an NHP of the species *C. penicillata* in an urban area of MOC city, Minas Gerais State, Brazil. In relation to the presence of rabies virus in frugivorous Chiroptera of the *Artibeus* genus, the first detection in MOC was made in 2009^17^.

Although both materials were positive for rabies virus in the molecular tests, it was not possible to establish the variant involved in these cases. The molecular results sent by the Pasteur Institute reported that the diagnosis of rabies was compatible with *D. rotundus* hematophagous bat. According to literature^4^, the most common variant of the rabies virus in *D. rotundus,* in Brazil is AgV3.

The relationship between humans and wild animals is becoming increasingly close, leading to synanthropism due to the expansion of cities towards areas of native vegetation. This causes environmental imbalances, forcing animals to adapt to urban environments and to search for food and shelter.

The confirmation of rabies in the animals in this study showed that the rabies virus is present and circulating in the urban area of MOC and serves as an alert about the importance of adopting permanent preventive measures to avoid rabies spillover to humans by *C. penicillata*, as previously reported for *C. jacchus* ^12^ ^,^ ^13^. Such measures include the annual antirabies vaccination of dogs and cats, avoiding physical contact with wild animals, and providing health education to prevent rabies infections in animals and humans. 

Therefore, this case report reinforces that physical contact with *C. penicillata* by the population of MOC city represents a real risk for contamination by the rabies virus, as previously noticed in humans who had contact with *C. jaccus* in Brazil^12^ ^,^ ^13^. 
